# Computational Investigations into Two-Photon Fibril Imaging Using the DANIR-2c Probe

**DOI:** 10.1021/acs.jpcb.2c07783

**Published:** 2023-04-04

**Authors:** N. Arul Murugan, Robert Zaleśny

**Affiliations:** † Department of Computational Biology, Indraprastha Institute of Information Technology, New Delhi 110020, India; ‡ Faculty of Chemistry, Wrocław University of Science and Technology, Wyb. Wyspiańskiego 27, PL-50370 Wrocław, Poland

## Abstract

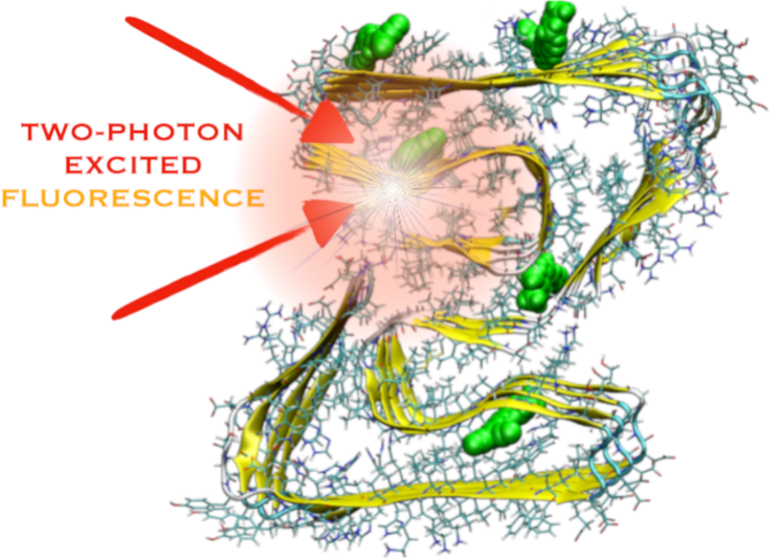

The design of novel fibril imaging molecules for medical diagnosis requires the simultaneous optimization of fibril-specific optical properties and binding specificity toward amyloid fibrils. Because of the possibility to monitor internal organs and deep tissues, the two-photon probes that can absorb in the infrared (IR) and near-IR (NIR) region with a significant two-photon absorption cross section are of immense interest. To contribute to this exploration of chemical compounds suitable for two-photon fibril imaging, we have computationally studied the one- and two-photon properties of a donor–acceptor-substituted DANIR-2c probe, which was used for in vivo detection of β-amyloid deposits using fluorescence spectroscopy. In particular, a multiscale computational approach was employed involving molecular docking, molecular dynamics, hybrid QM/MM molecular dynamics, and coupled-cluster/MM to study the binding of the studied probe to amyloid fibril and its one- and two-photon absorption properties in the fibrillar environment. Multiple binding sites are available for this probe in amyloid fibril, and the one corresponding to the largest binding affinity exhibits also the largest and experimentally meaningful two-photon absorption cross section, thus demonstrating the potential of the studied probe in two-photon microscopy.

## Introduction

Alzheimer’s disease and many other neurodegenerative diseases are some of the most devastating diseases to human health and are contributing to a significant world economic burden due to nonavailability of curative medicines. The currently available drugs approved by the US Food and Drug Administration (FDA), such as Donepezil, Rivastigmine, Galantamine, Memantine and Suvorexant, do not reverse the disease condition but treat cognitive and noncognitive symptoms associated with the disease. Even for diagnosis there are only a few tracers available, namely ^18^F-Florbetaben (Neuraceq), ^18^F-Florbetapir (Amyvid), ^18^F-Flutemetamol (Vizamyl), and ^18^F-Flortaucipir (Tauvid). The former three compounds were approved for positron emission tomography (PET)-based amyloid imaging while the last compound is recommended for PET imaging of tau fibrils in the brain. Early diagnosis can contribute to achieve better rehabilitation and improve the lifestyle of affected patients, and so the design of novel diagnostic agents having superior binding specificity for amyloid and tau fibrils is a research area of immense interest. Alzheimer’s disease is often associated with accumulation of amyloid and tau fibrils in the human brain, and a number of molecules for in vivo and in vitro have been proposed. The accumulation of tau fibrils can also be associated with other subgroup of neurodegenerative diseases termed tauopathies. However, the microstructures of tau fibrils are reported to be very different, and binding site microenvironments vary in size and in nature depending upon the specific class of tauopathies. Therefore, designing imaging agents with remarkable binding specificity is needed, which is one of the challenges in diagnostics development for Alzheimer’s-like neurodegenerative diseases. In the case of in vivo imaging agents, a radiolabeled probe molecule is injected into human subjects, and the emitting positron radiation provides the spatial (distribution and intensity of accumulation) nature of amyloid or tau fibrils in the brain region which can be directly used to infer about the disease condition. In the case of in vitro fibril imaging, the samples containing amyloid fibrils are treated/titrated with optical probe molecules whose absorption spectra and fluorescence spectra are dependent on the amyloid concentration (for example, Thioflavin-T and Congo Red-like molecules) and so can be used to estimate the fibrils quantitatively. In both cases the binding affinity and binding specificity are the most important properties to be optimized. The fibril-specific one-photon absorption spectra, fluorescence spectra, and two-photon absorption spectra are the other most relevant properties to be considered in the case of in vitro optical probes but do not have any significance in the case of in vivo imaging applications. However, any optical probes used for in vitro imaging can be converted to an in vivo PET tracer by suitably attaching a radio label, and this may of course require expertise in substitution reaction chemistry. The message is that any progress made in the development of novel optical probes for fibril imaging can also contribute to development of tracers suitable for in vivo PET imaging of fibrils. The cost involved in developing and validating such optical probes for fibril imaging is much cheaper when compared to that corresponding to in vivo tracer development. In addition, two-photon probes for fibril monitoring have enormous application in the validation of drug candidates in animal models where it is required to monitor the fibril accumulation in the brain during different stages of drug trials. Two-photon imaging can be a potential tool in mouse models as the effect of drugs on the accumulation of fibrils in the brain of living subjects can be monitored effectively.^1,2^ For example, a two-photon probe SAD1 has been used to monitor the amyloid beta deposits below 380 μm depths in living transgenic mice.^3^

Even though a plethora of one-photon-absorption- and fluorescence-based optical probes for fibril imaging are available,^4−7^ the number of two-photon probes is very limited. As far as we know, there are only a few molecules reported to be suitable for two-photon fibril imaging. For example, LDS821,^8^ TZPI,^9^ STB8,^10^ DCPI,^11^ SAD1,^3^ CRANAD-3,^1,2^ and DANIR-OH 2b as well as DANIR-OH 2c^12^ are some of the two-photon probes shown to be suitable for fibril imaging. As we know the size of the chemical library of organic compounds (with molecular mass below 500 Da)^13^ is on the order of 10^60^, but for two-photon fibril imaging application we have only a few molecules from such a huge chemical space. It clearly indicates that the compounds from different chemical libraries need to be screened either experimentally or computationally to identify novel molecules for fibril imaging. As the experimental setup associated with two-photon imaging is very expensive, it is rational to perform such screening using computational approaches. However, the suitability of currently available methods for estimating the binding affinity and one- and two-photon absorption properties of molecules in fibril-like environment need to be tested. Even though the computational approaches were often shown successful in computing the optical and magnetic properties in a vacuum and solvents, the modeling in heterogeneous environments like fibrils, enzymes, membranes, DNA, RNA, and quadruplex in solvents is considerably challenging and requires use of a multiscale multiphysics approach. We can only see a limited number of studies that focus on modeling the two-photon properties of molecules in biomolecular environments such as membranes and enzymes.^14−19^

**Scheme 1:**
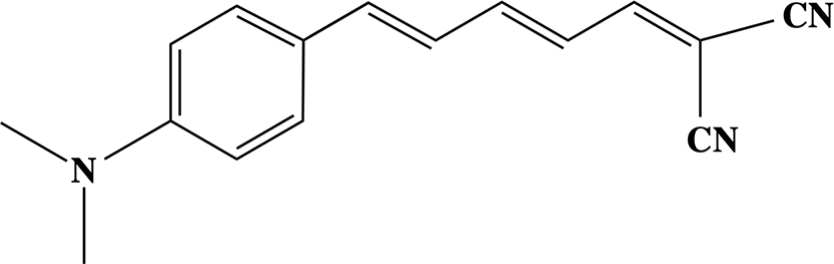
DANIR-2c Molecule Studied in This Work

In this work, we use a multiscale multiphysics approach to study the binding affinity as well as one- and two-photon properties of the DANIR-2c molecule in solvent- and fibril-like environments. This molecule has already been successfully applied in detection of β-amyloid deposits, but using conventional fluorescence techniques.^20^ Thus, given the experimentally confirmed efficiency of DANIR-2c for fibril imaging, we assess its potential for two-photon imaging. To that end, we have used an integrated approach involving molecular docking, molecular dynamics, hybrid QM/MM molecular dynamics, and RI-CC/MM for modeling these properties. In particular, the binding sites and modes of DANIR-2c in fibrils were identified from molecular docking, while the binding affinities in different sites were estimated using the molecular mechanics-generalized Born surface area approach, an implicit solvent free-energy calculation method. For this, 1000 configurations from molecular dynamics trajectories were used. Furthermore, the hybrid QM/MM molecular dynamics has been performed to study the molecular structure and electronic structure of DANIR-2 in different binding sites of amyloid fibril. Finally, for various configurations picked up from the hybrid QM/MM MD, the RI-CC2/MM calculations were performed to compute the fibril specific one- and two-photon absorption spectra.

## Computational Details

**Figure 1:**
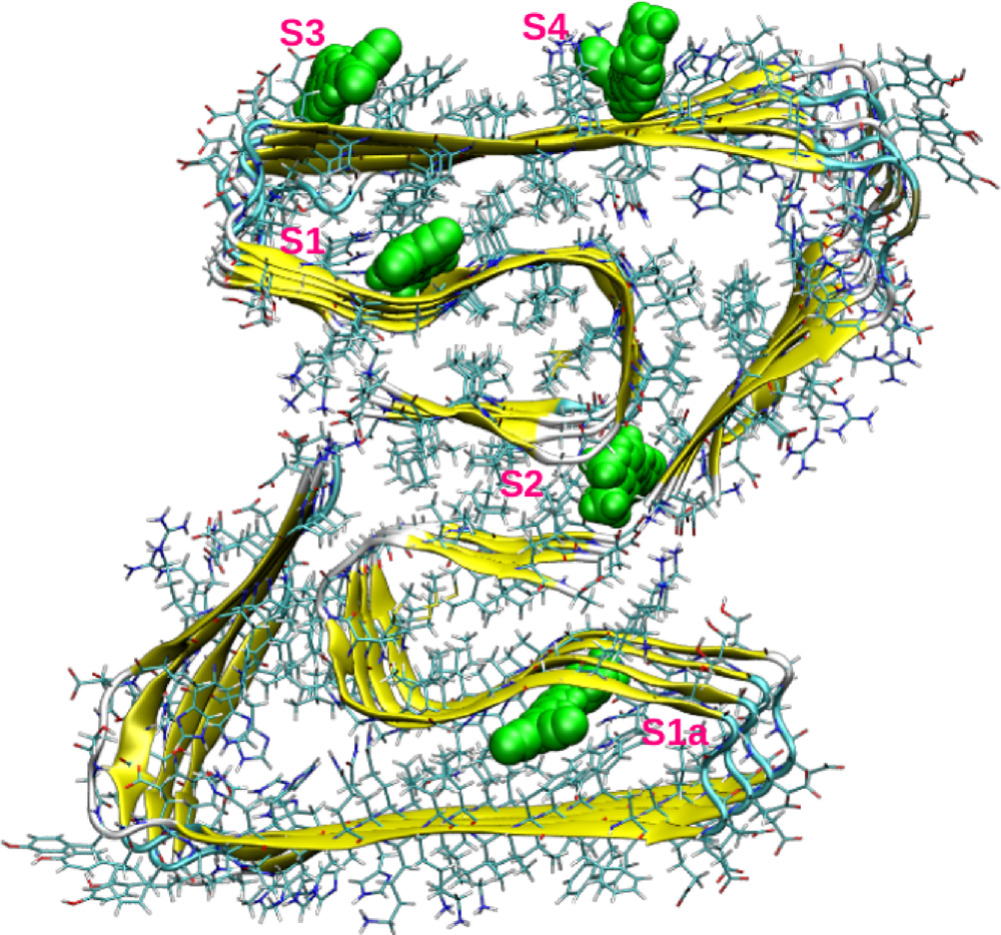
Different binding sites for DANIR-2c in the amyloid fibril.

The molecular docking is the approach in general used to identify the binding sites for chemical compounds in different target biomolecules. Here, we performed the molecular docking using Autodock4.0 software.^21^ The amyloid fibril structure used in this study is based on the cryogenic-electronic microscopy as reported in the protein database with reference ID 5OQV.^22^ The structure includes the two interwined protofilaments made of tetramer and pentamer of two amyloid beta (1–42) fibrils. Furthermore, it provides the coordinates for the whole range of amino acids, i.e., 1–42, and so serves as one of the most relevant target structures for structure-based computational study. Certain studies included only pentamer units of protofibrils and so ignored the sites in the interfacial region.^23−25^ Here we include the entire protofibril, and so the molecular docking algorithm could locate binding sites on the surface, in the core, and in the interfacial region (see Figure 1). As the binding site details for DANIR-2c are not available, we performed a blind docking by including the entire fibril within the grid box. The number of grids was chosen appropriately (number of grid points along *x*, *y*, and *z* directions were 220, 200, and 130 with a default grid size of 0.375 Å). Initially, the molecular structure of DANIR-2c was optimized using the B3LYP/6-31+G* level of theory as implemented in Gaussian09 software,^26^ and the optimized molecular structure has been used for the molecular docking. The Antechamber^27^ (as available in Ambertools)^28^ and AutodockTools^21^ module were used to convert the Gaussian output files to PDBQT format which is required for carrying out the molecular docking. The Gasteiger-type charges were added during this conversion. The molecular docking finds various possible binding sites in the fibril, and the most stable binding modes in different sites are identified. We have used the Lamarckian genetic algorithm to identify the most stable binding sites and binding modes for DANIR-2c within the fibril. In particular, the algorithm samples over the translational, rotational, and conformational/torsional degrees of freedom of the ligands while the fibril target is treated as a rigid body. The docking energies (binding affinities) are estimated for each of the conformations, and the algorithm finds a number of most stable structures for the ligands within the binding site. The DANIR-2c molecule is found to be binding to 2 surface sites, 2 interfacial sites, and 1 core site within the fibril. The fibril structure with five DANIR-2c molecules bound to different binding sites (in their most stable binding modes) has been considered as the starting configuration for the subsequent molecular dynamics and hybrid QM/MM molecular dynamics simulations. This is computationally less demanding when compared to carrying out different MD simulations for each binding mode of DANIR-2c in the fibril.

In the case of molecular dynamics simulations, we used FF14SB,^29^ general AMBER force-field (GAFF),^30^ and TIP3P^31^ force fields for amyloid fibril, DANIR-2c, and water, respectively. The charges for amyloid and water subsystems are already provided in the force field, which is not the case for the DANIR-2c molecule. In the case of DANIR-2c in various binding sites, the charges were computed by fitting to the molcular electrostatic potential computed using the B3LYP/6-31G(d) level of theory, and the fitting procedure termed CHELPG^32^ is implemented in Gaussian09 software. The complex has been solvated and neutralized by adding sufficient number of counterions (in this case 27 Na^+^ ions were added as each amyloid beta (1–42) peptide chain carried −3*e* charges). The simulations involved minimization runs as well as simulation in a constant volume ensemble and in an isothermal isobaric ensemble. The time step for the integration of equation of motion was 2 fs, and the time scale for equilibration run was about 5 ns. The production run in isothermal–isobaric ensemble was performed for a time scale of 100 ns. The 1000 configurations from the last 10 ns trajectory was used for the calculation of binding free energies using the molecular mechanics-generalized Born surface area (MM-GBSA) approach.^33^ The molecular dynamics simulations were performed using Amber20 software^34^ while the MM-GBSA binding free energy calculations were performed using the MMPBSA.py module^35^ available in AmberTools.

The final structure (DANIR-2c bound to different binding sites in amyloid fibril) as obtained from the equilibration run of molecular dynamics simulation has been used as the input structure for Car–Parrinello hybrid QM/MM molecular dynamics.^36^ Similar to force-field MD, this also involves minimization. Subsequently, the temperature scaling run and finite temperature run in constant volume ensemble using a Nosé–Hoover thermostat were used. There were five independent QM/MM simulations corresponding to DANIR-2c in different binding sites of amyloid fibrils. As the ligands are bound to binding sites that are spatially separated, it is not possible to describe all the ligands as a QM subsystem in a single simulation run. In these simulations, DANIR-2c was treated using the BLYP level of theory, and the rest of the systems (remaining four DANIR-2c ligands, fibrils, ions, and solvents) were treated using the force field. In this implementation, the QM system is described using a plane-wave-based wave function, and 80 Ry has been used as the energy cutoff. In the QM/MM molecular dynamics simulation, the electrostatic and van der Waals interaction between QM and MM subsystems are accounted for using an effective QM/MM Hamiltonian. The QM subsystem is polarized by the effective charges in MM subsystems, and the environment-induced changes in the molecular and electronic structure of the ligand are accounted for in this approach. We have reported that this is an advantage with the use of hybrid QM/MM MD when compared to force-field MD as these structural changes are very important for modeling the biomolecule specific optical (one-photon, two-photon, and hyperpolarizabilities) properties in the ligands.^37−39^ The time step for the integration of the equation of motion was 5 au, and the calculations were performed for a total time scale of 100 ps. The calculations were performed using CPMD and GROMOS software,^40−43^ which respectively treat the dynamics of QM and MM subsystems. The electrostatic embedding between these two subsystems was described using the interface codes which are distributed with GROMOS software.^42,43^ 100 independent configurations from the Car–Parrinello molecular dynamics simulations were used for computing one- and two-photon absorption spectra using RI-CC2/MM theory as implemented in the TURBOMOLE program.^44,45^

The absorption spectra and two-photon absorption cross sections for DANIR-2c in fibrils were computed by employing quadratic response theory^44,46,47^ combined with the CC2 method and the TZVP basis set of Ahlrichs et al.^48^ Within the RI-CC2 non-Hermitian (NH) response theory framework, the rotationally averaged two-photon transition strengths for the |0⟩ → |*J*⟩ transition, in the case of a single beam of linearly polarized monochromatic light, are given by^47^
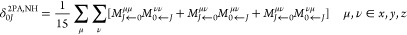
1*M*_*J*←0_^μν^ and *M*_0←*J*_^μν^ denote the μν-th component of the right and left second-order transition moments, respectively. The sum-over-states expressions for the transition moments are given by
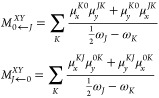
2where ω_*K*_ represents the excitation energy for the |0⟩ → |*K*⟩ transition and μ_*x*_^*KL*^ = ⟨*K*|*X*|*L*⟩ in eq 2 is the *x*-component of the first-order transition dipole moment for the |*K*⟩ → |*L*⟩ transition. The superscripts on μ distinguish the right (*L*0) and left (0*L*) first-order transition moments. The two-photon absorption cross section was calculated based on the following formula:^49^
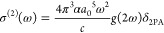
3where *g*(2ω) is the line shape function, *a*_0_ is the Bohr radius, α is the fine structure constant, and *c* is the speed of the light. In order to determine two-photon absorption cross section at absorption band maxima, we averaged δ^2PA^ values close to absorption band maximum in the range Δ*E*_ave_ ± ε, where ε = 0.05 eV. The line shape function *g*(2ω) was represented by the Gaussian profile, and FWHM was determined based on statistical analysis of probe-fibril configurations for each binding site. All RI-CC2 calculations, including parameters entering generalized few-state model (see next section), were performed using the TURBOMOLE program.^44,45^

## Results and Discussion

The binding free energies and different contributions such as van der Waals, electrostatic, and polar and nonpolar solvation energies are given in Table 1. The free energies were computed as average over 1000 configurations corresponding to last 10 ns trajectory from the production run. As reported in previous studies,^37,50^ DANIR-2c shows varying binding affinities for different binding sites. The core site (site 1) is the one associated with the larger binding affinity (associated with a binding free energy of −43.4 kcal/mol), which is also the case for a number of tracers and optical probes binding to amyloid fibrils.^50^ The binding affinity for site 1a (refer to Figure 1) which is symmetrically the same also has comparable binding affinity (i.e., −42.6 kcal/mol) to that of site 1. The interfacial binding site (termed site 2 in Figure 1), which is located between the two filaments (made of pentamer and tetramer), is the next high affinity binding site (associated with binding free energy corresponding to −25.1 kcal/mol). The surface binding sites have low binding affinities, and in particular DANIR-2c in site 3 dissociates during the coarse of the simulation which is also evident from the lower binding affinity (refer to Table 1). The second surface binding site (termed site 4) has comparable binding free energies to that of the interfacial binding site. Experimentally, it was shown that the DANIR-2c has superior binding affinity for amyloid fibril than the popular amyloid staining probe, Thioflavin-T (ThT).^20,51^ Consistent with the report, the binding free energies computed for DANIR-2c are lower than those reported for ThT previously.^50^

**Table 1:** Binding Free Energies and Contributions from van der Waals, Electrostatic, Polar, and Nonpolar Solvation Free Energies^a^

system	Δ*E*_vdw_	Δ*E*_elec_	Δ*G*_GB_	Δ*G*_SA_	Δ*G*_binding_
site 1	–54.4	–28.3	45.3	–6.0	–43.4(±0.1)
site 2	–41.1	–37.6	58.6	–5.0	–25.1(±0.1)
site 3	–13.5	–10.0	15.8	–1.9	–9.7(±0.2)
site 4	–31.9	–21.4	35.2	–4.0	–22.1(±0.1)

a The free energies were computed using the MM-GBSA approach, and the energies are in kcal/mol.

**Figure 2:**
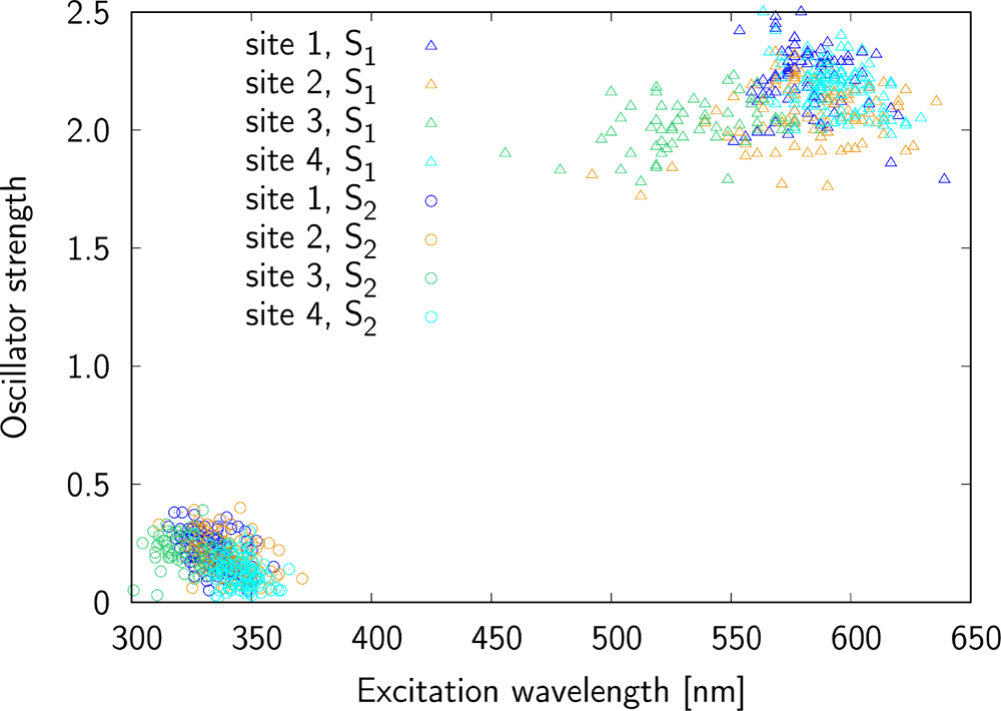
One-photon S_0_ → S_1_ and S_0_ → S_2_ excitations for sites 1–4.

**Figure 3:**
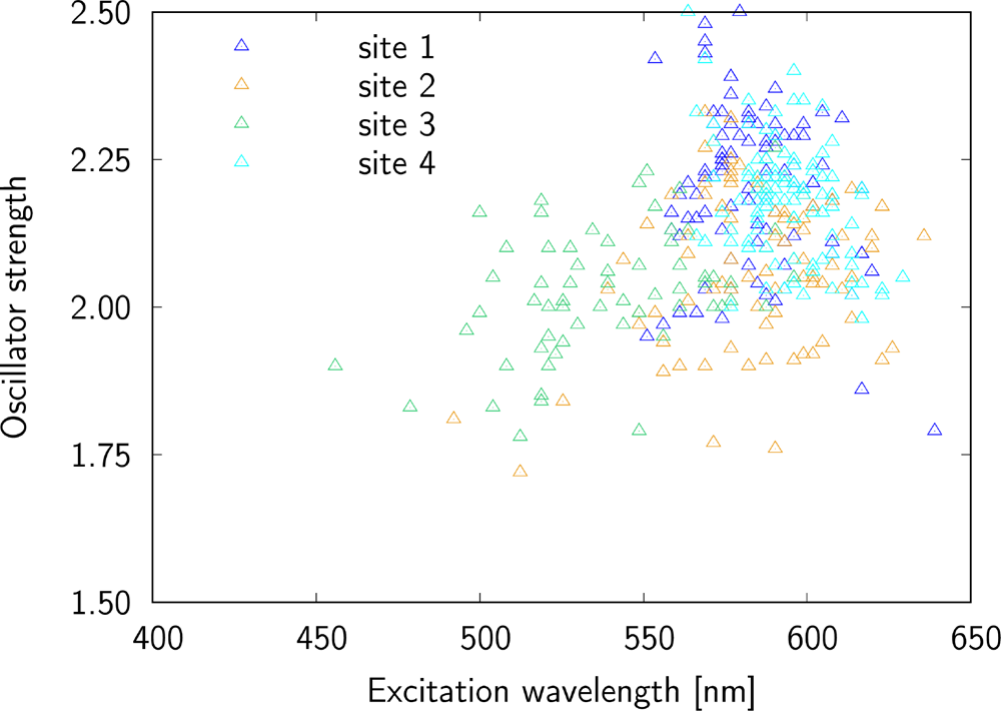
One-photon S_0_ → S_1_ excitation for sites 1–4.

As already mentioned in the preceding section, we used the RI-CC2 method and electrostatic embedding approach to determine site-dependent one-photon excitation wavelength and the corresponding oscillator strengths. The results of these calculations are presented in Figures 2 and 3 (the Supporting Information contains complementary figures for individual sites; see Figures S1–S6). Several conclusions can be drawn from Figures 2 and 3. First, the S_1_ and S_2_ electronic excited states are well separated, regardless of the binding site; i.e., the differences between average excitation wavelengths for these two electronic states are 249, 243, 214, and 249 nm for site 1, site 2, site 3, and site 4, respectively. Second, the nature of the two states is quite different as demonstrated by the quite different distribution of excitation wavelengths, which is much higher for the S_1_ excited state hinting charge-transfer excitation susceptible to local microenvironment. The differences between maximal and minimal wavelengths corresponding to the S_0_ → S_1_ transition are the largest for site 2 (144 nm) and site 3 (140 nm), while for the remaining sites these differences do not exceed 90 nm. Third, the most red-shifted average value of excitation wavelength is found for site 4 (594 nm), while the most blue-shifted average wavelength is for site 3 (538 nm).

**Figure 4:**
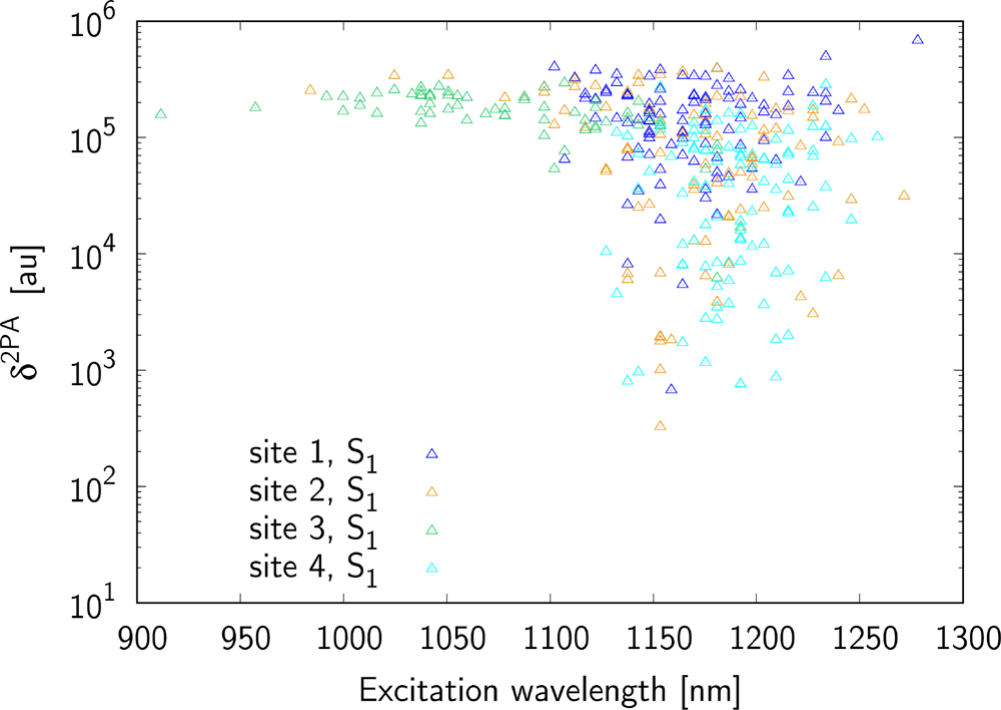
Two-photon S_0_ → S_1_ excitations for sites 1–4.

Given the low excitation wavelength corresponding to S_0_ → S_2_ transition, which makes it unsuitable for two-photon imaging, we will analyze two-photon absorption properties for excitation to the lowest electronic excited state. Figure 4 shows site-specific two-photon transition strength, which is purely molecular parameter. Clearly, there is a wide span of the values covering range from 10^3^ to 10^5^ a.u. (with only a few outliers below 10^3^ a.u.). The mean values of two-photon transition strengths determined close to absorption band maxima are 1.68 × 10^5^ a.u. (site 1), 0.88 × 10^5^ a.u. (site 2), 1.79 × 10^5^ a.u. (site 3), and 0.46 × 10^5^ a.u. (site 4) These values correspond to significant two-photon absorption cross sections: 1178 GM (site 1), 392 GM (site 2), 711 GM (site 3), and 378 GM (site 4). As highlighted by Kim et al., the product of two-photon absorption cross section and fluorescence quantum yield should exceed 50 GM to make a molecule an effective probe for bioimaging.^52^ As seen from the above data, the probe binding is the largest in site 1, and this corresponds to the largest two-photon absorption cross section. Moreover, in all three cases the two-photon absorption band maxima fall in the range 1073 nm (site 3) to 1188 nm (site 4), which fits into the desired biological transparency window.

**Figure 5:**
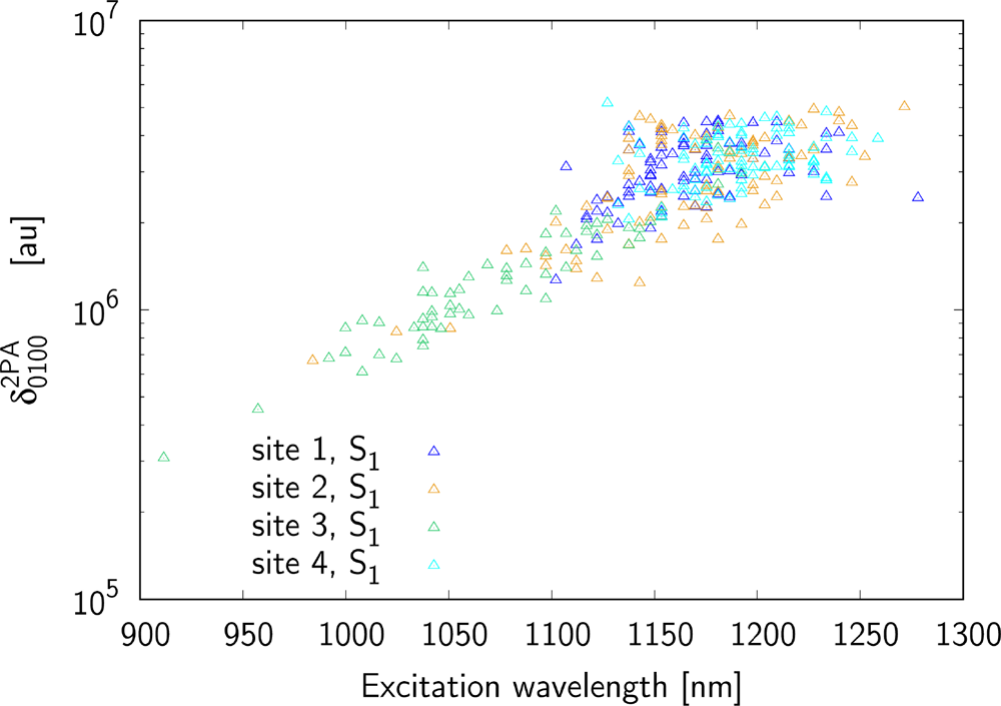
Contribution from δ_0100_^2*PA*^ term to the two-photon S_0_ → S_1_ excitation for sites 1–4.

**Figure 6:**
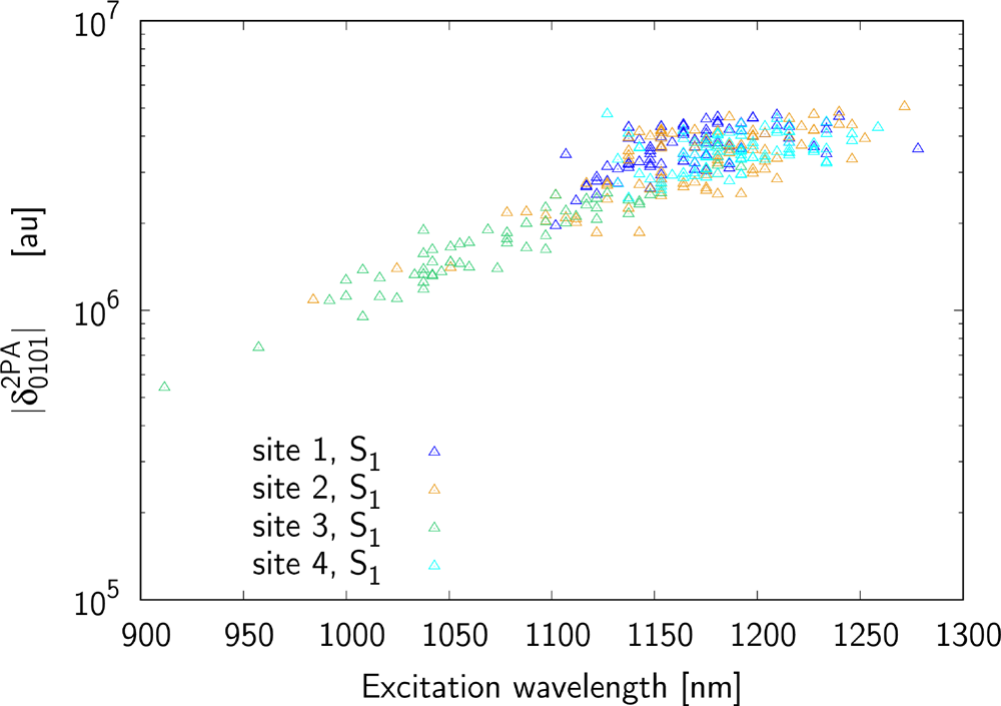
Contribution from δ_0101_^2*PA*^ term to the two-photon S_0_ → S_1_ excitation for sites 1–4 (note that absolute value is shown).

**Figure 7:**
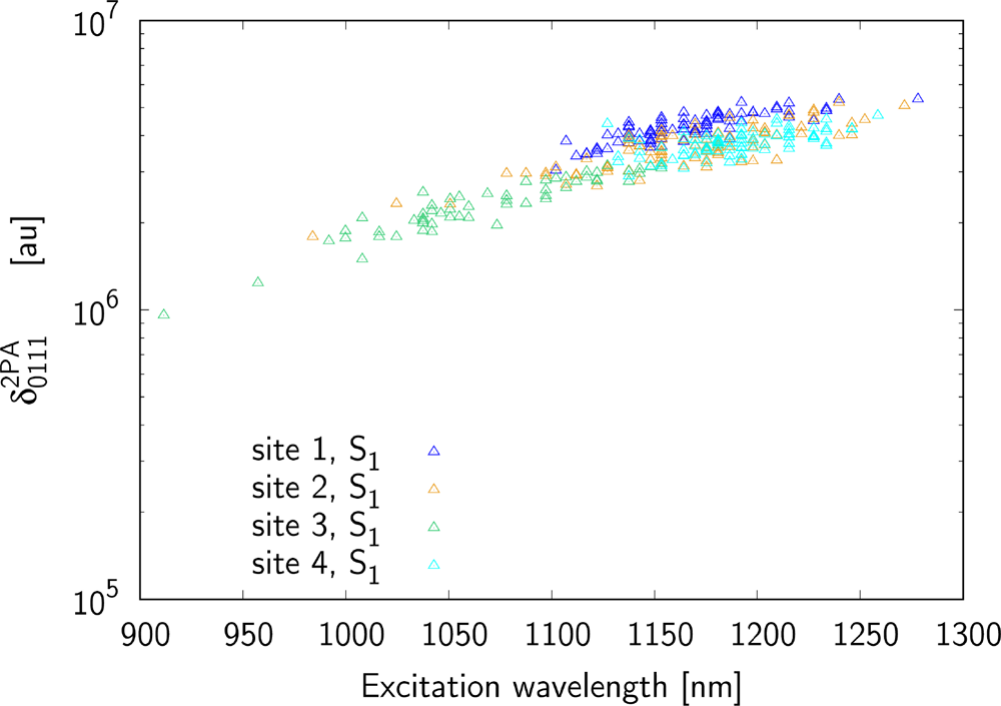
Contribution from δ_0111_^2*PA*^ term to the two-photon S_0_ → S_1_ excitation for sites 1–4.

In order to gain an insight into the site-specific fluctuations in two-photon transition strengths (and corresponding two-photon absorption cross sections), we have employed the generalized few-state model.^53,54^ Starting with eq 1, it is possible to separate the magnitude of the transition moments from the angle (orientation) terms, thus leading to the GFSM expressions.^53,54^ The final equation for the 2PA strength for a GFSM for the non-Hermitian theories is^54^
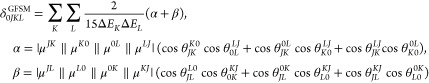
4In eq 4,  and the term θ_*PQ*_^*RS*^ represents the angle between the transition dipole moment vectors μ_*PQ*_ and μ_*RS*_. Expressions for different few-state models can be obtained from eq 4 by choosing a given number of intermediate states *K* and *L*. For a two-state model (2SM), as used in this work, *K* and *L* can be either the ground state 0 or the final excited state *J*. The sum over *K* and *L* thus reduces to four terms: δ_0*J*00_, δ_0*J*0*J*_ ≡ δ_0*JJ*0_ and δ_0*JJJ*_. Figures 5–7 present the terms contributing to the S_0_ → S_1_ two-photon transition strengths under a two-state approximation. It should be noted that all four terms are similar in terms of magnitude; however, the two identical terms δ_0110_^2*PA*^ and δ_0101_^2*PA*^ are negative (for the sake of clarity, shown are absolute values of these two terms on a logarithmic scale). In the case of the majority of studied snapshots, the following pattern holds:
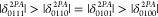
The largest term, δ_0111_^2*PA*^, is a product of |μ^01^∥μ^10^∥μ^11^|^2^ so the two-photon S_0_ → S_1_ transition is governed by the dipole moment in the S_1_ excited state and one-photon S_0_ → S_1_ transition intensity. We note that this term shows the smallest variations among all studied terms, and this is particularly true in the case of site 1. Presumably, this is the explanation of largest two-photon cross section values for this site.

## Conclusions

Even though the chemical space of organic molecules is huge, the number of molecular probes available for two-photon imaging of fibrils is very low. The candidate molecules should possess binding specifity for amyloid fibrils and considerable two-photon absorption cross sections in their fibril-bound conformation. The multiphysics study performed using molecular docking, molecular dynamics, hybrid QM/MM molecular dynamics, and RI-CC/MM approaches clearly demonstrates that DANIR-2c can be a potential molecular probe for two-photon imaging of fibrils. The molecule is shown to have high binding affinity for amyloid fibrils (better than ThT, a known popular amyloid staining molecule), and in addition it exhibits the two-photon absorption cross section much larger than 100 GM, which is the threshold required for imaging (assuming fluorescence quantum yield equal to 0.5). Interestingly, the two-photon absorption cross sections appear to be binding site dependent, and its maximum is found for site 1 which exhibits the largest binding affinity for DANIR-2c. The use of integrated computational approach allows for identification of novel molecules for two-photon imaging of amyloid and tau fibrils. This can contribute to the validation of promising compounds for the two-photon imaging studies of amyloids, which are one of the causative factors responsible for Alzheimer’s disease, as this technique facilitates the temporal and spatial estimation of amyloids in the brain of living animal (a mouse in particular) models.

## Supporting Information Available

Electronic one- and two-photon absorption spectra at different binding sites (PDF)
