# Supplementary material for: Computational Investigations into Two-Photon Fibril Imaging Using the DANIR-2c Probe
Source: J Phys Chem B. 2023 Apr 4;127(14):3119–25. doi: 10.1021/acs.jpcb.2c07783 (PMC10108348; doi:10.1021/acs.jpcb.2c07783)
Supplement: Supplementary file 1 — jp2c07783_si_001.pdf [file jp2c07783_si_001.pdf]

Supporting Information: Computational Investigations into Two-Photon Fibril Imaging Using DANIR-2c Probe

N. Arul Murugan*,† and Robert Zaleśny*,‡

†*Department of Computational Biology, Indraprastha Institute of Information Technology, New Delhi, India*

‡*Faculty of Chemistry, Wrocław University of Science and Technology, Wyb. Wyspiańskiego 27, PL–50370 Wrocław, Poland*

E-mail: arul.murugan@iiitd.ac.in; robert.zalesny@pwr.edu.pl

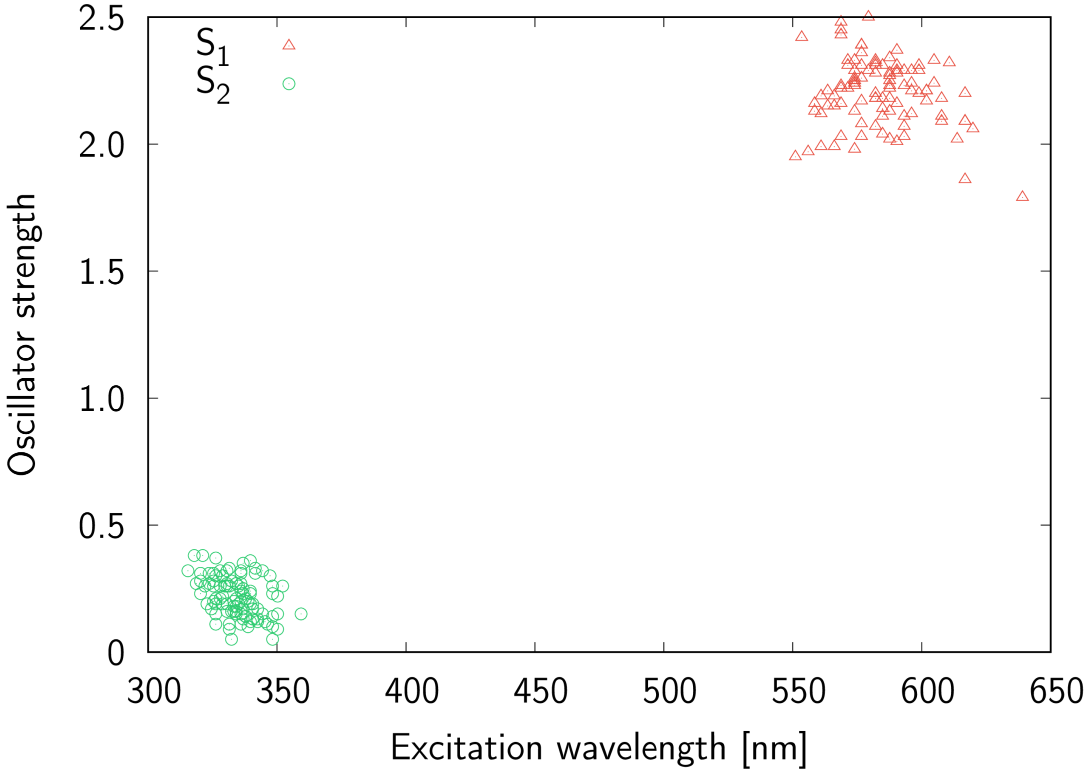

Figure S1: One-photon $S_0 \rightarrow S_1$ and $S_0 \rightarrow S_2$ excitations for **site 1**

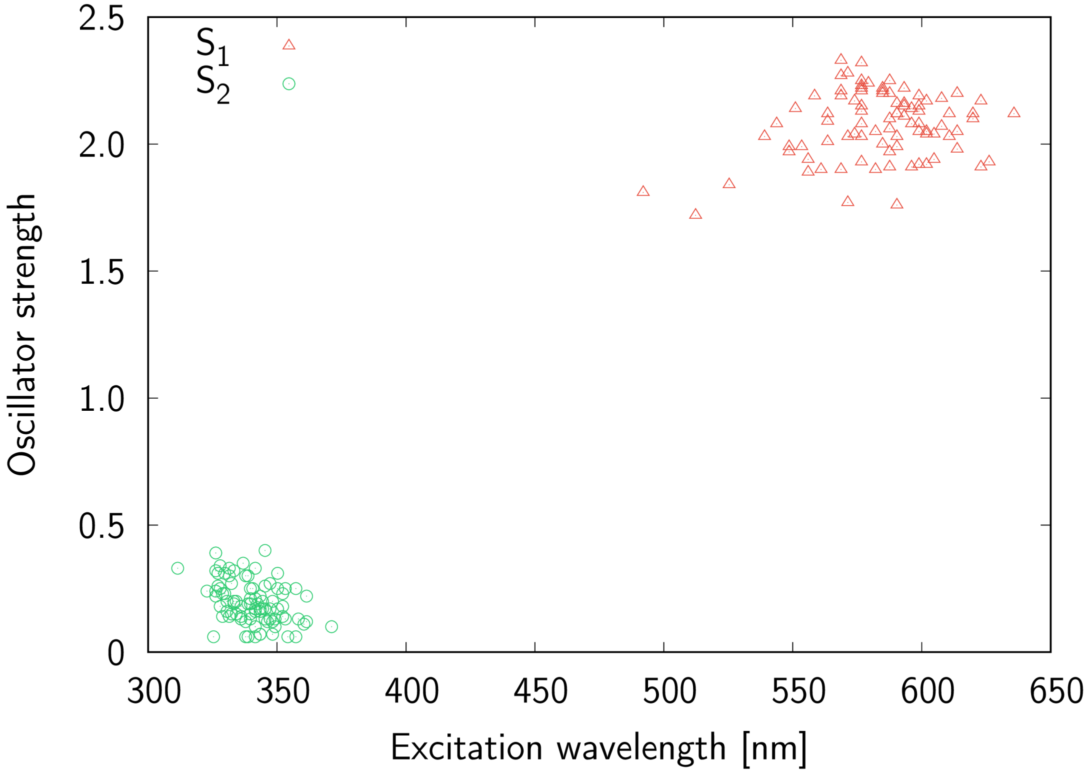

Figure S2: One-photon $S_0 \rightarrow S_1$ and $S_0 \rightarrow S_2$ excitations for **site 2**

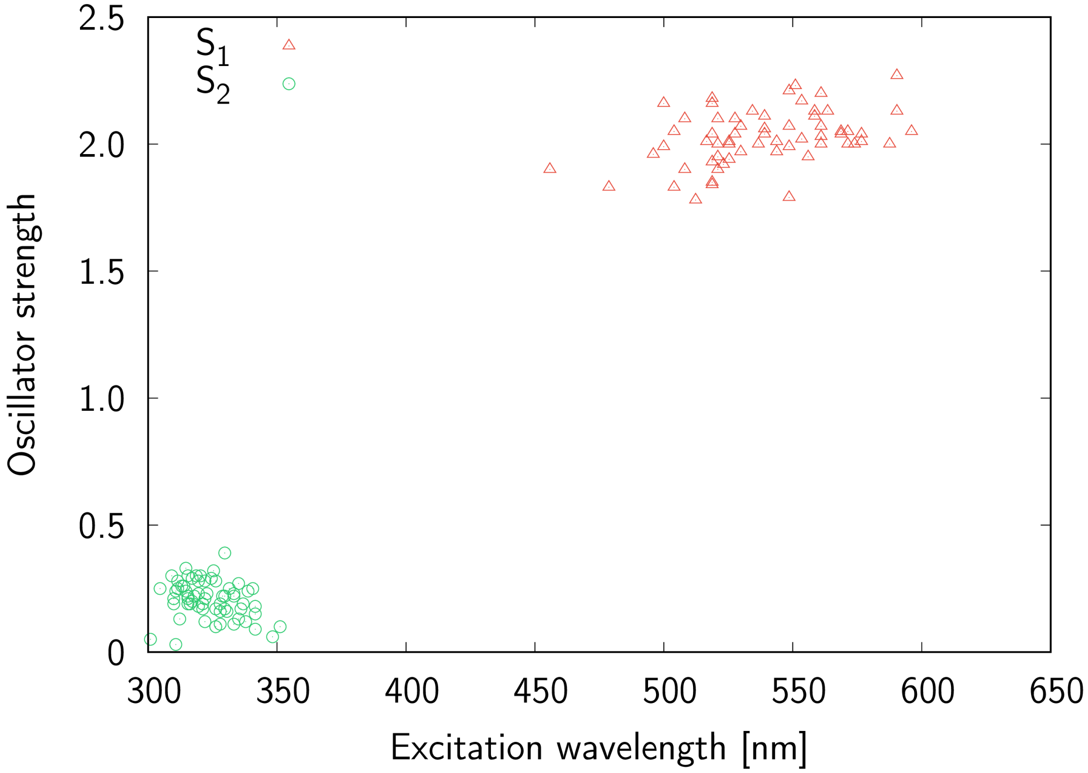

Figure S3: One-photon $S_0 \rightarrow S_1$ and $S_0 \rightarrow S_2$ excitations for **site 3**

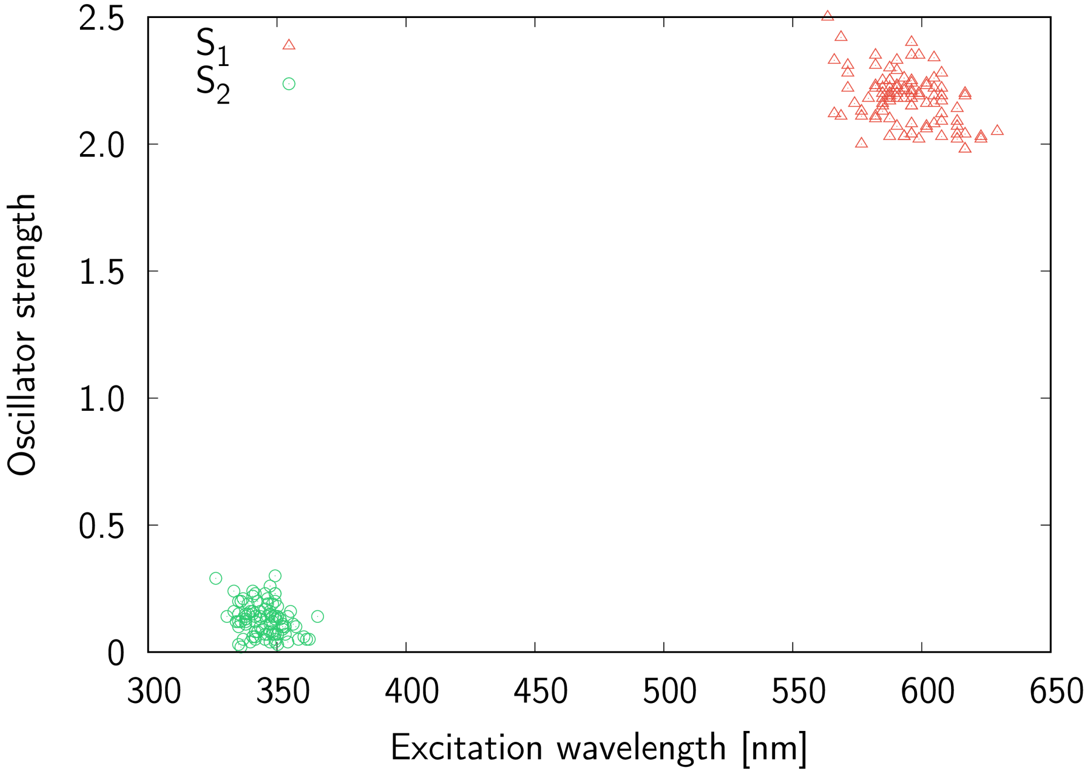

Figure S4: One-photon $S_0 \rightarrow S_1$ and $S_0 \rightarrow S_2$ excitations for **site 4**

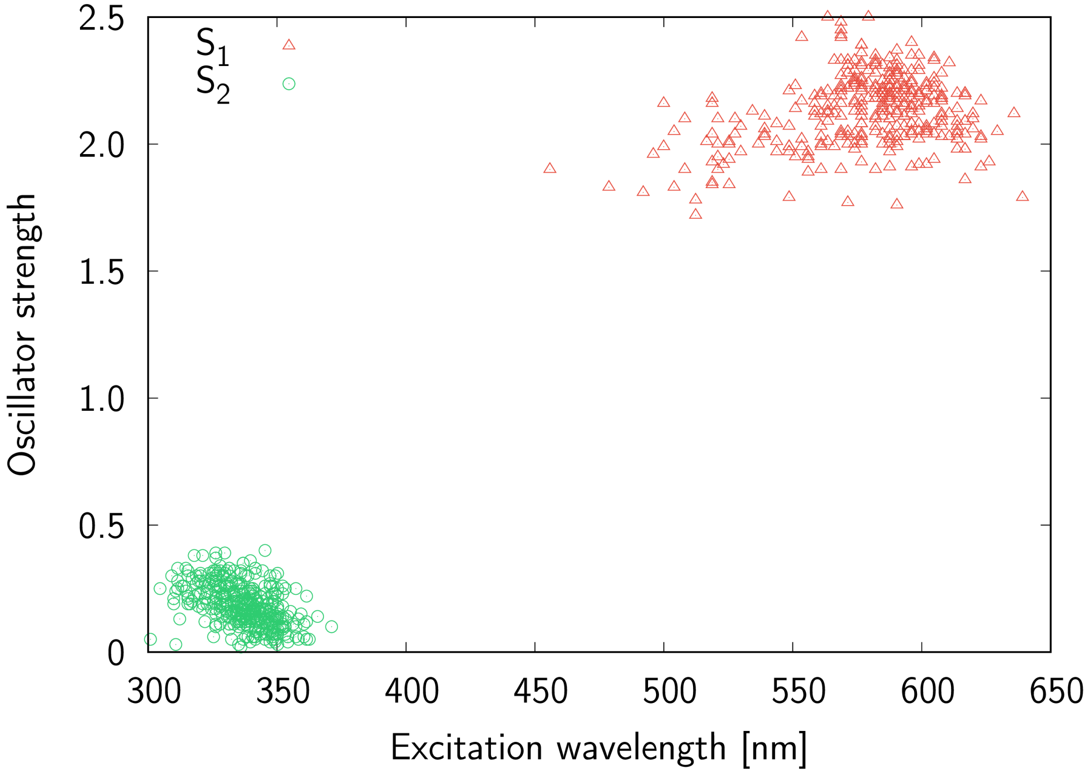

Figure S5: One-photon $S_0 \rightarrow S_1$ and $S_0 \rightarrow S_2$ excitations for **sites 1–4**

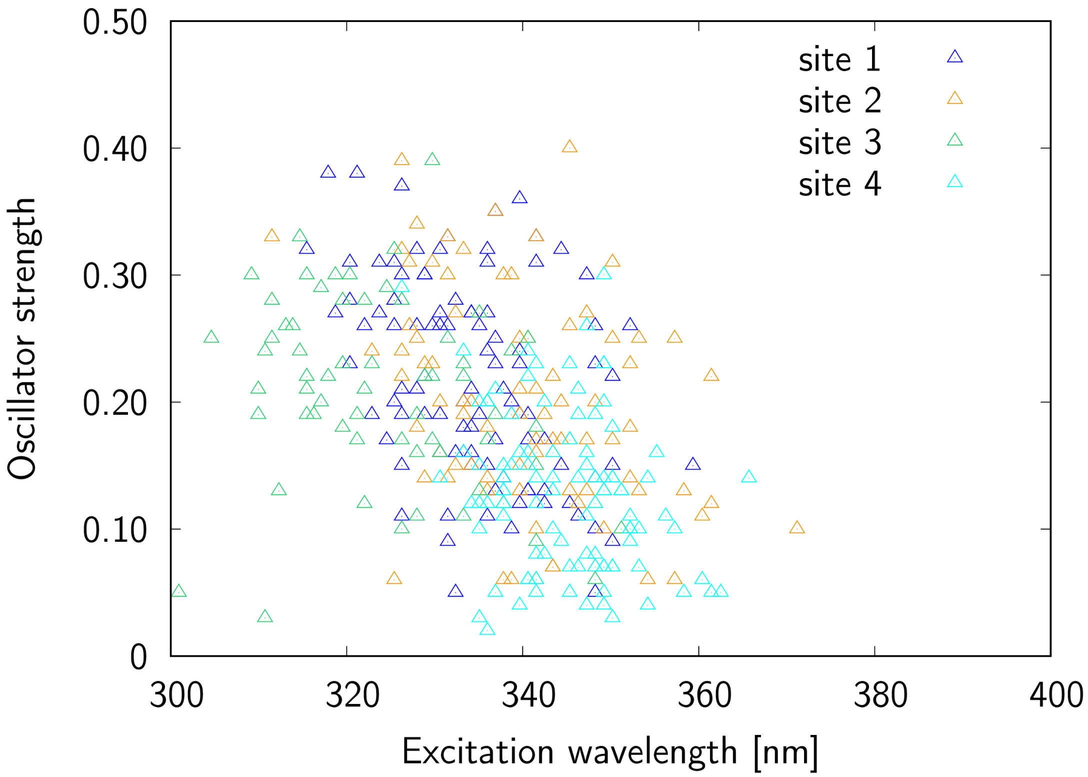

Figure S6: One-photon $S_0 \rightarrow S_2$ excitation for **sites 1–4**

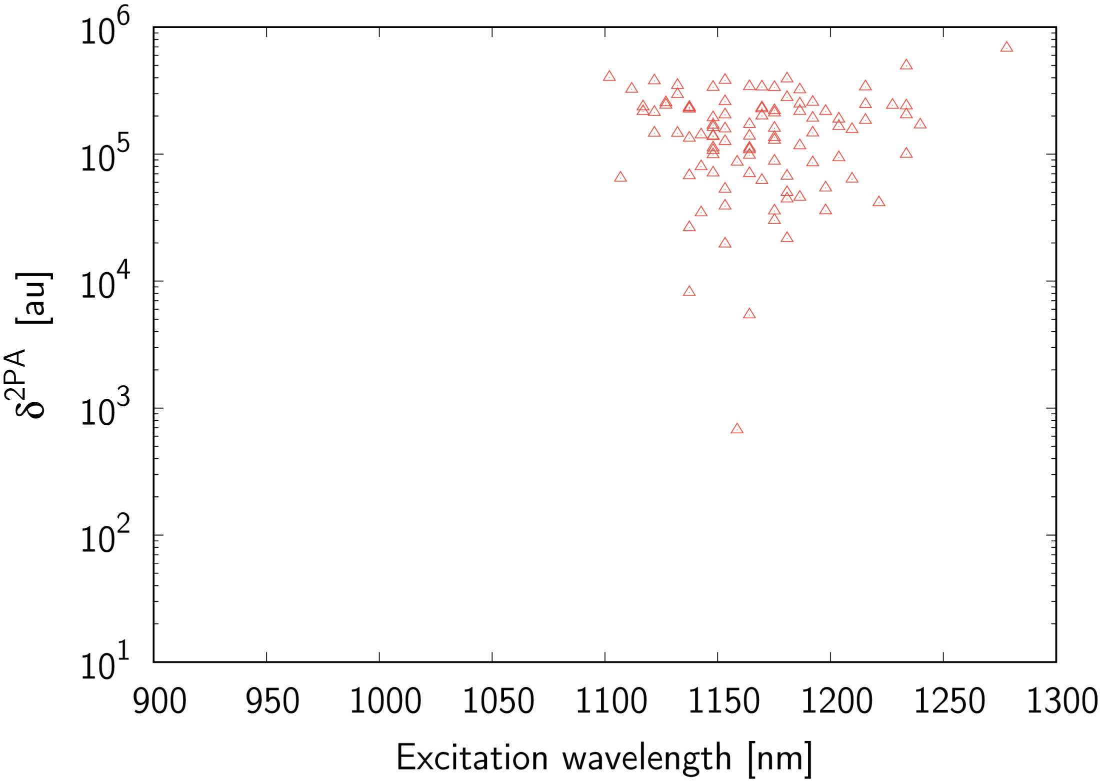

Figure S7: Two-photon S_0 $\rightarrow S_1$ excitation for **site 1**

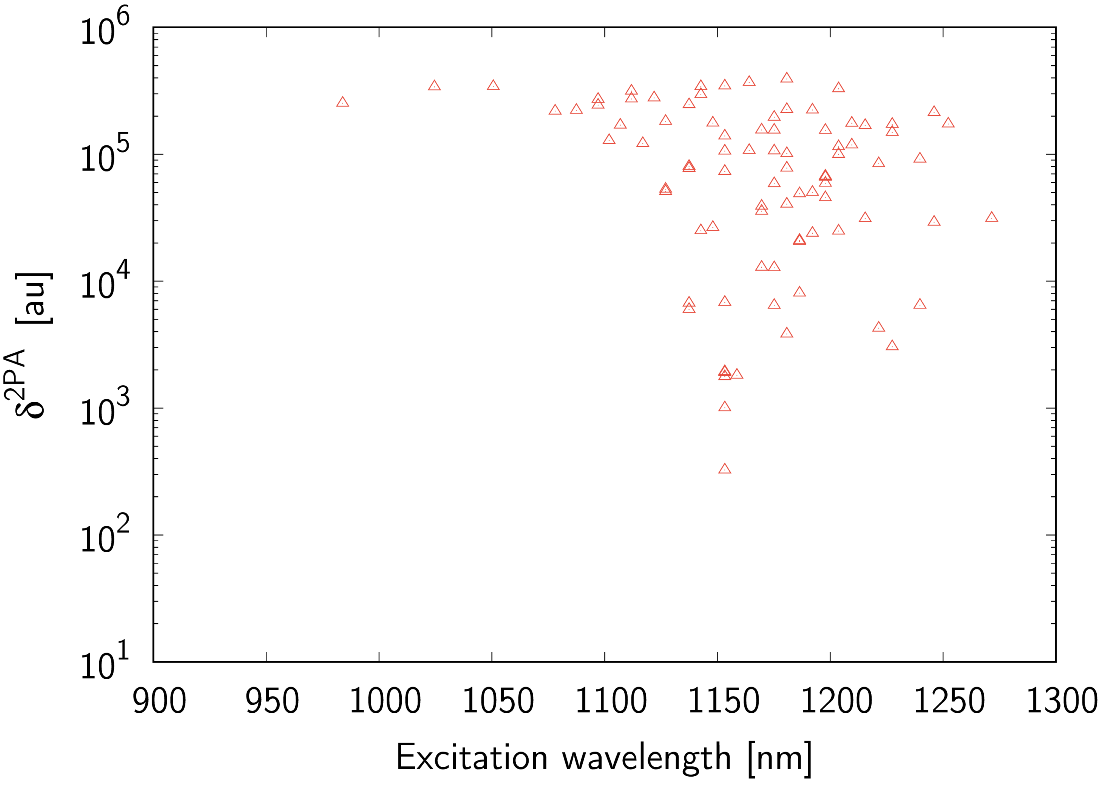

Figure S8: Two-photon $S_0 \rightarrow S_1$ excitation for **site 2**

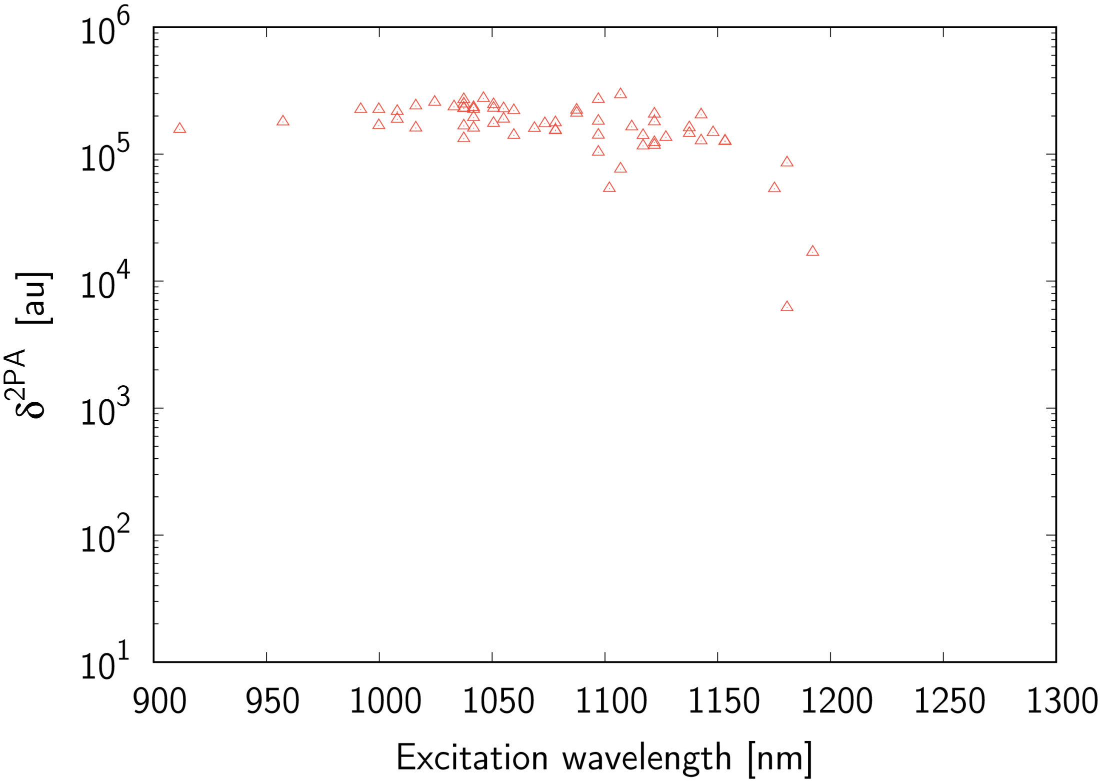

Figure S9: Two-photon $S_0 \rightarrow S_1$ excitation for **site 3**

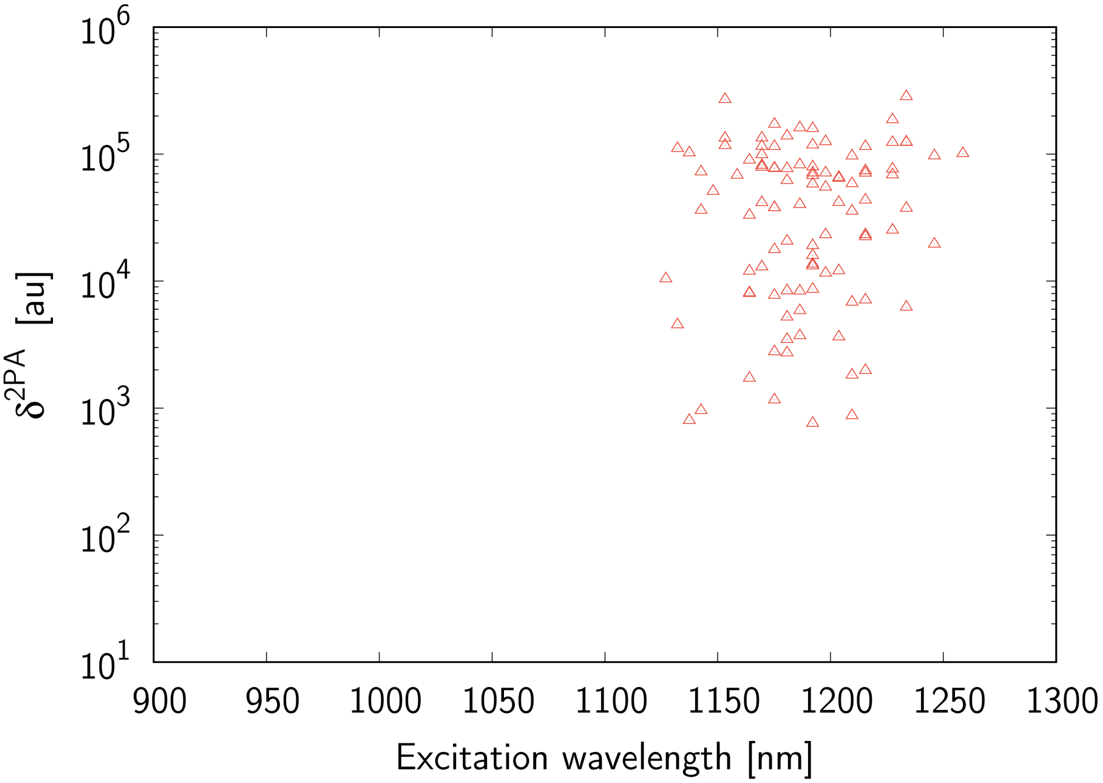

Figure S10: Two-photon $S_0 \rightarrow S_1$ excitation for **site 4**

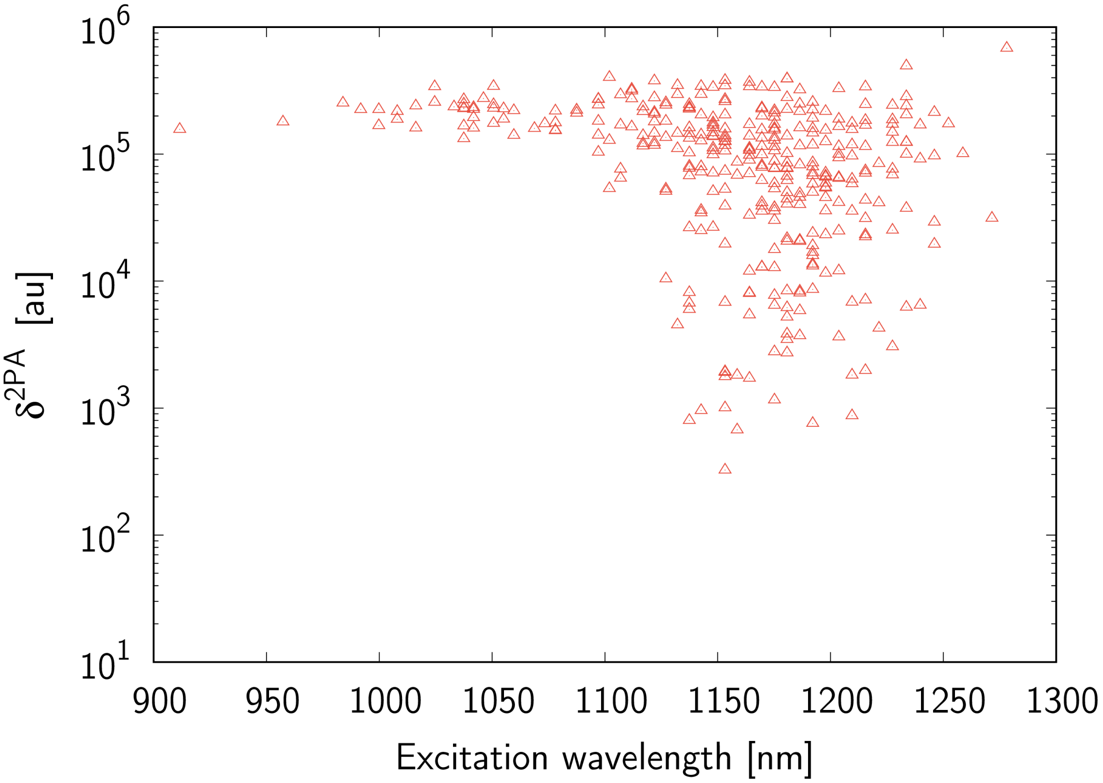

Figure S11: Two-photon $S_0 \rightarrow S_1$ excitations for **sites 1–4**

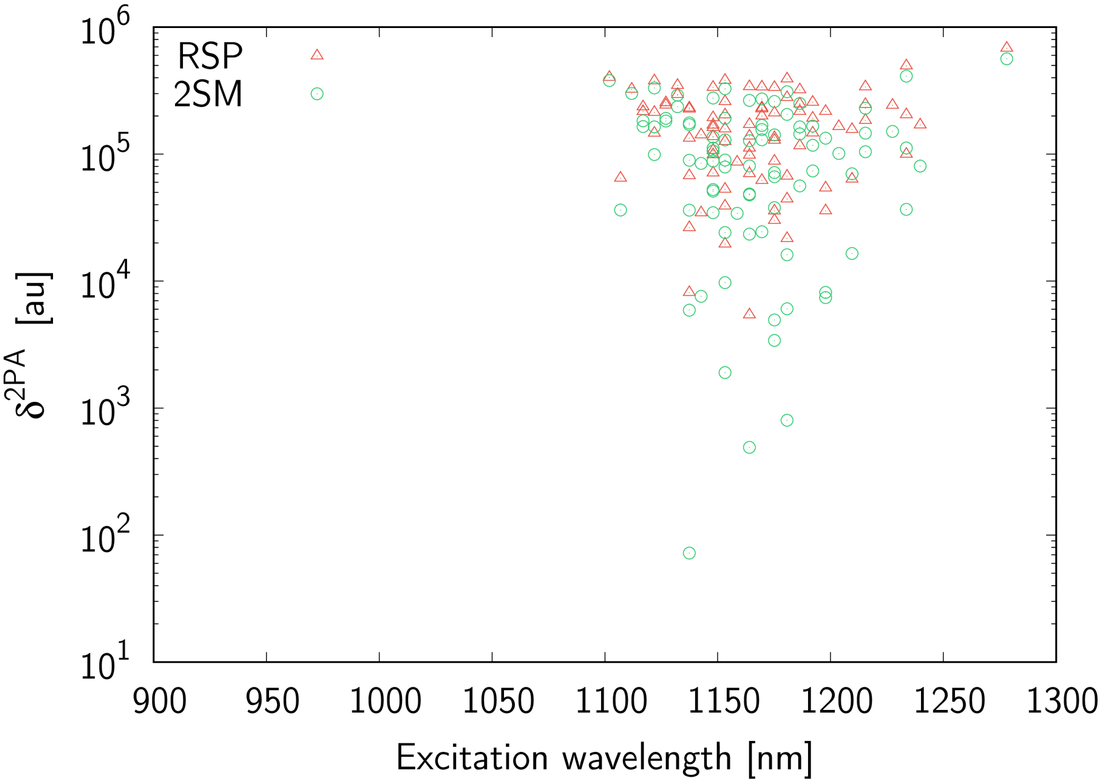

Figure S12: Two-photon $S_0 \rightarrow S_1$ excitation for **site 1**

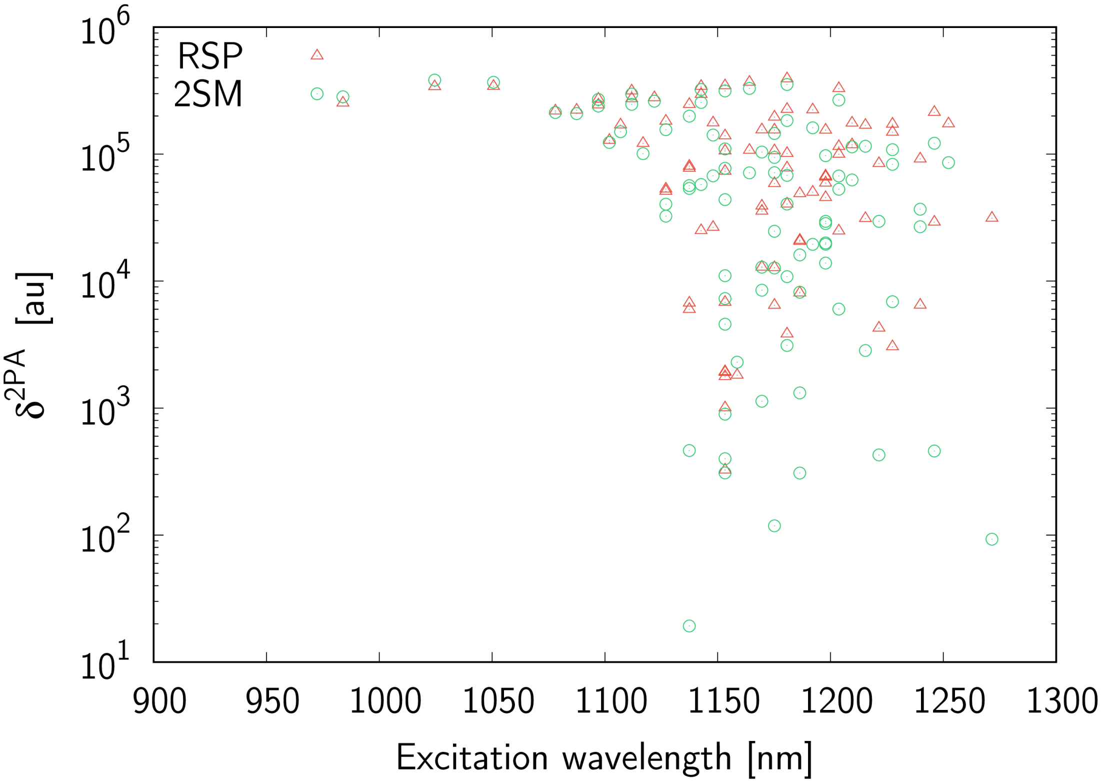

Figure S13: Two-photon $S_0 \rightarrow S_1$ excitation for **site 2**

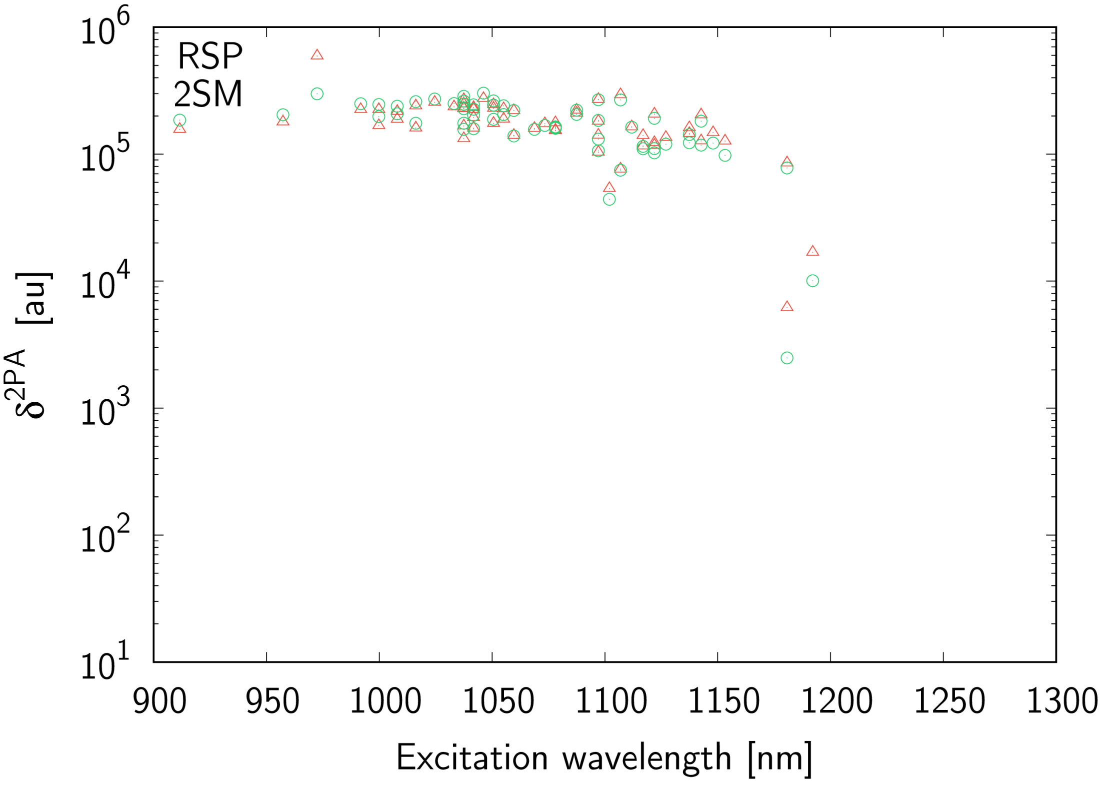

Figure S14: Two-photon $S_0 \rightarrow S_1$ excitation for **site 3**

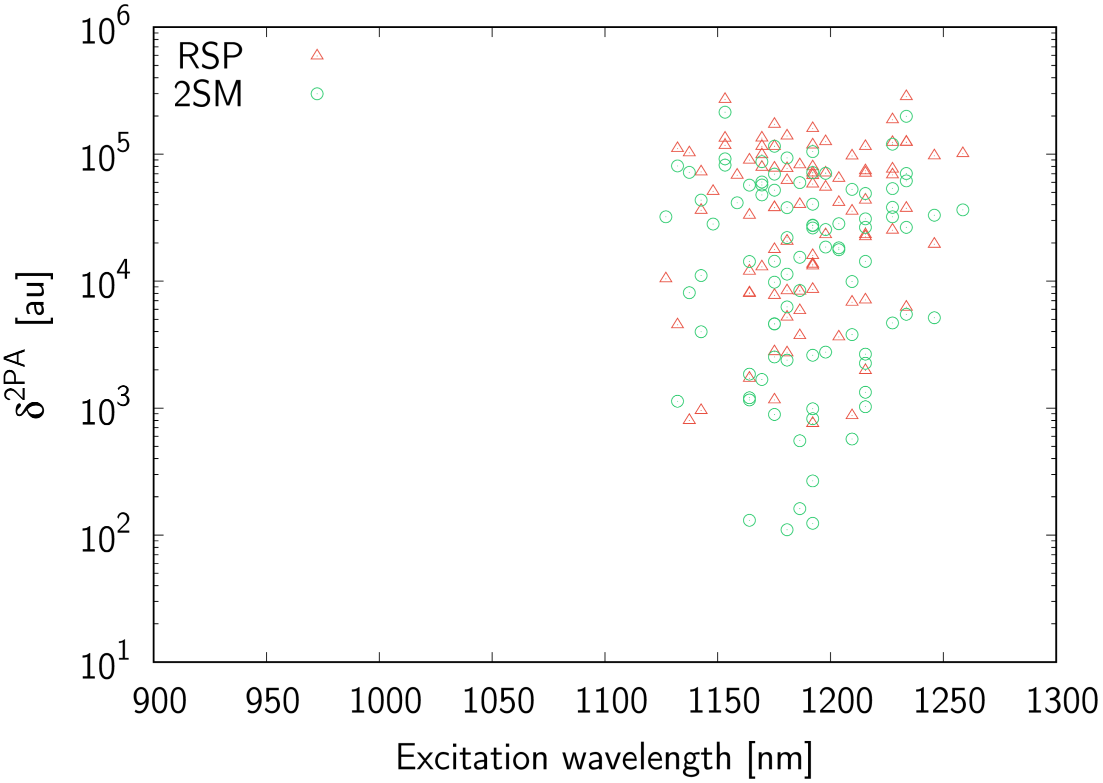

Figure S15: Two-photon $S_0 \rightarrow S_1$ excitation for **site 4**
